# Phylogeny of *Morella rubra* and Its Relatives (Myricaceae) and Genetic Resources of Chinese Bayberry Using RAD Sequencing

**DOI:** 10.1371/journal.pone.0139840

**Published:** 2015-10-02

**Authors:** Luxian Liu, Xinjie Jin, Nan Chen, Xian Li, Pan Li, Chengxin Fu

**Affiliations:** 1 Key Laboratory of Conservation Biology for Endangered Wildlife of the Ministry of Education, and Laboratory of Systematic & Evolutionary Botany and Biodiversity, College of Life Sciences, Zhejiang University, Hangzhou 310058, China; 2 Laboratory of Fruit Quality Biology/The State Agriculture Ministry Laboratory of Horticultural Plant Growth, Development and Quality Improvement, Zhejiang University, Hangzhou 310058, China; Saint Mary's University, CANADA

## Abstract

Phylogenetic relationships among Chinese species of *Morella* (Myricaceae) are unresolved. Here, we use restriction site-associated DNA sequencing (RAD-seq) to identify candidate loci that will help in determining phylogenetic relationships among *Morella rubra*, *M*. *adenophora*, *M*. *nana* and *M*. *esculenta*. Three methods for inferring phylogeny, maximum parsimony (MP), maximum likelihood (ML) and Bayesian concordance, were applied to data sets including as many as 4253 RAD loci with 8360 parsimony informative variable sites. All three methods significantly favored the topology of (((*M*. *rubra*, *M*. *adenophora*), *M*. *nana*), *M*. *esculenta*). Two species from North America (*M*. *cerifera* and *M*. *pensylvanica*) were placed as sister to the four Chinese species. According to BEAST analysis, we deduced speciation of *M*. *rubra* to be at about the Miocene-Pliocene boundary (5.28 Ma). Intraspecific divergence in *M*. *rubra* occurred in the late Pliocene (3.39 Ma). From pooled data, we assembled 29378, 21902 and 23552 de novo contigs with an average length of 229, 234 and 234 bp for *M*. *rubra*, *M*. *nana* and *M*. *esculenta* respectively. The contigs were used to investigate functional classification of RAD tags in a BLASTX search. Additionally, we identified 3808 unlinked SNP sites across the four populations of *M*. *rubra* and discovered genes associated with fruit ripening and senescence, fruit quality and disease/defense metabolism based on KEGG database.

## Introduction

Many domesticated fruit trees, such as peach (*Prunus persica* (L.) Batsch), plum (*Prunus salicina* Lindl.), kiwifruit (*Actinidia deliciosa* (A. Chev.) C. F. Liang & A. R. Ferguson and *A*. *chinensis* Planch) and persimmon (*Diospyros kaki* L. f.) originated in China [1–3]. Among them, some were derived from a single wild species, while others involved multiple species. In addition, some fruits even experienced a much more complicated domestication process. For example, Xu et al. [4] presented evidence to suggest that sweet orange [*Citrus sinensis* (L.) Osbeck] originated from a backcross hybrid between pummelo [*C*. *grandis* (L.) Osbeck] and mandarin (*C*. *reticulata* Blanco). Regardless of how domestication occurred, an unambiguous phylogeny of domesticated species and their potential source is always helpful for understanding the processes involved. Chinese bayberry is one of the most popular fruits in southern China. It is generally thought to have been domesticated from the wild *M*. *rubra* Lour., a subtropical evergreen tree with a wide distribution in China, Japan, Korea and the Philippines [5–6]. The domestication process has remained unresolved because the phylogenetic relationships among *M*. *rubra* and its relatives are still unclear.

The Myricaceae is a small, sub-cosmopolitan family of about 50 species, and the family of predominantly shrubs and trees are characterized by unisexual flowers borne in catkins, peltate glands, entire leaves, a unilocular ovary and single orthotropous ovule. With the exception of two monotypic genera, *Comptonia* L'Hér. ex Ait. and *Canacomyrica* Guillaumin, the species of Myricaceae have traditionally been referred to the Linnaean genus *Myrica* [7]. However, *Myrica* was split into two genera, *Myrica sensu stricto* and *Morella* Lour., based on morphological differences (deciduous or evergreen; dry fruits or fleshy fruits; sunken stoma or not) and phylogenetic analysis of nuclear ITS and chloroplast *trnL-F* sequence data [8]. In Myricaceae, only one genus and four species are in China; *M*. *rubra*, *M*. *nana* (A. Chev.) J. Herb, *M*. *adenophora* (Hance) J. Herb. and *M*. *esculenta* (Buch.-Ham. ex D.Don) I. M. Turner. *M*. *esculenta* and *M*. *adenophora* are easily recognized by their distinct tomentose branchlets and petioles, while *M*. *rubra* and *M*. *nana* are glabrous or sparsely pubescent [9]. Previous phylogenetic studies [8] strongly supported the monophyly of these four species, but the relationships among *M*. *rubra*, *M*. *nana* and *M*. *adenophora* were not resolved. The genetic diversity and population structure of wild *M*. *rubra* populations are poorly known. Insufficient knowledge of phylogenetics and population genetics will hinder the improvement of Chinese bayberry cultivars and the breeding of new ones.

Phylogeny reconstruction within closely related species may be difficult because of incomplete lineage sorting, introgression, short evolutionary scale, and lack of molecular markers in poorly studied taxa [10]. In this circumstance, reduced-representation genome sequencing methods allow us to sequence the regions flanking restriction sites with deep coverage, then to align orthologous sequences across multiple samples to discover thousands of genetic markers for systematics, population genomics and adaptive evolution studies [11–14]. These methods, including restriction site associated DNA sequencing (RAD-seq) and genotyping by sequencing (GBS), are promising and can be easily applied to non-model organisms with no reference genome sequence [15–16]. Hitherto, RAD-seq has been successfully applied to phylogenetic inference in *Pedicularis* [15], temperate bamboos [17], as well as population genomics in *Lagenaria siceraria* (Molina) Standl. [18], *Gasterosteus aculeatus* [19], and adaptive evolution in *Entosphenus tridentatus* [20], *Myodes glareolus* [16].

In Zhejiang, Fujian and Guangdong provinces, Chinese bayberry is one of the most popular and valuable fruits because of its appealing color, delicious taste and essential micronutrients [21]. The fruit is not only eaten fresh, dried and canned, but is also widely used for making wine and juice [22]. It also exhibits a wide range of pharmacological properties due to the high content of anthocyanins, which are reported to have anti-inflammatory, anti-tumor and anti-oxidative properties [23]. According to the literature, Chinese bayberry has been cultivated for more than 2000 years in southern China, and the cultivated area is currently 340,000 ha, with an annual yield of 1.1 million tons valued at 1.5 billion dollars [24]. It has long been a major source of income for farmers in some counties. During the long history of cultivation, more than one hundred cultivars, such as ‘Dongkui’, ‘Biqi’ and ‘Wandao’, were developed [25]. In recent years, Chinese bayberry has been exported to foreign countries and has received international attention due to its extraordinary qualities [26]. However, the development of Chinese bayberry is now confronted by huge challenges. Firstly, there is no consensus on the classification of cultivars, and they are classified only according to ripening date, fruit color, fruit weight and kernel characteristics, resulting in a high frequency of synonyms and homonyms [27]. Secondly, fruit quality declines rapidly at room temperature, which leads to a short shelf life [28–29]. Moreover, with the increase in planting area, Chinese bayberry suffers from a range of pathogens [such as *Phomopsis myricina* Y. J. Huang et P. K. Chi and *Leptographium abietinum* (Peck) M. J. Wingfield] [30–31]. Previous studies attempted to regulate fruit quality during ripening, to control postharvest fruit decay [32–33] and to enhance pathogen resistance [34]. However, knowledge of wild germplasm resources, phylogeny and the molecular basis of fruit quality and defense from disease is limited. The above problems will seriously inhibit the future development of the Chinese bayberry industry.

The objectives of the present study are 1) to resolve the phylogenetic relationships among *M*. *rubra* and its relatives, 2) to discover SNP markers within populations of *M*. *rubra* for future studies on genetic diversity and population structure, and 3) to determine if several genes or pathways are associated with fruit ripening and senescence, fruit quality and disease resistance.

## Materials and Methods

### Ethics statement

Three individuals from North America were permitted by Harvard University Herbaria (USBH) and NCSU Herbaria (USR). Management Bureau of Mt. Gutian National Nature Reserve issued the permit for Gutian Mountain (ZJGT); Management Bureau of Mt. Leigong National Nature Reserve issued the permit for Leigong Mountain (GZLS); Kunming Forestry Bureau issued the permit for sampling in Kunming (YNFM, YNXW, YNZJ, YNPL, YNHX and YNAL). No specific permissions were required for other locations which are neither privately owned nor protected and the field study did not involve endangered or protected species.

### Sample collection and DNA extraction

**Fig 1:**
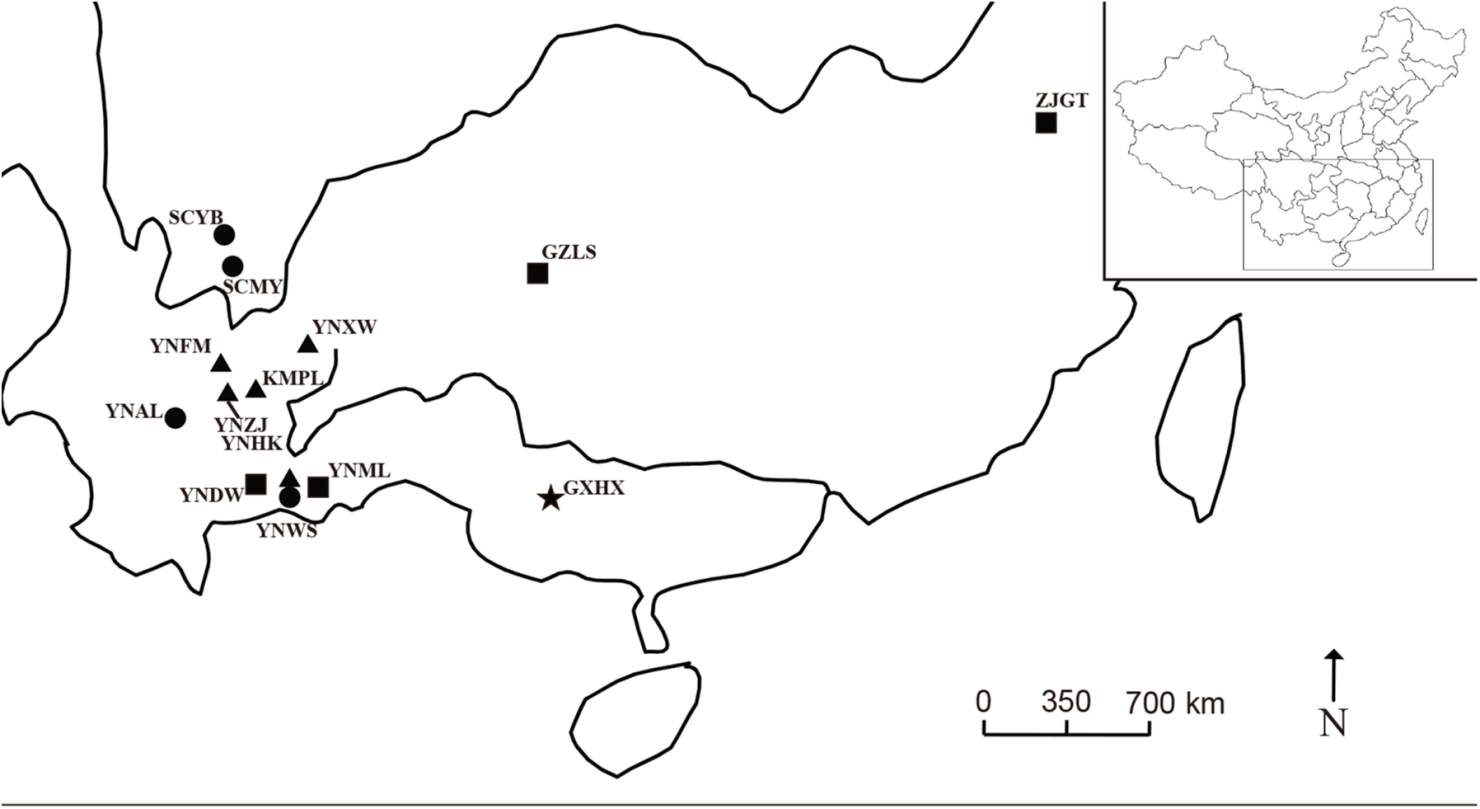
Map of sampling locations of *Morella* in China. Squares indicate *Morella rubra*; triangles indicate *M*. *nana*; dots indicate *M*. *esculenta*; star indicates *M*. *adenophora*.

Eighteen individuals, including six species of *Morella* as well as the closely related outgroup species, *Comptonia peregrine* (L.) Coult., were collected between 2012 and 2014 in China and North America (Table 1, Fig 1). The ingroup samples included one individual of *M*. *cerifera* (L.) Small, one of *M*. *pensylvanica* (Mirb.) Kartesz, four of *M*. *esculenta*, six of *M*. *nana*, one of *M*. *adenophora* and four of *M*. *rubra*. Total genomic DNA was extracted from silica-dried leaf tissue using the modified protocol of Doyle [35].

**Table 1:** Details of location and sampling information for species of *Morella* investigated in this study.

Taxon	ID	Location	Latitude	Longitude	Altitude(m)
*Morella rubra*	ZJGT	Gutianshan, Zhejiang, China	N29°8’41.77"	E118°9’11.18"	455
	GZLS	Leigongshan, Guizhou, China	N26°30’1.04"	E106°44’2.82"	1135
	YNDW	Daweishan, Yunnan, China	N22°55’29.21"	E103°40’46.38"	2257
	YNML	Malipo, Yunnan, China	N23°8’16.67"	E104°41’23.71"	1171
*M*. *adenophora*	GXHX	Hengxian, Guangxi, China	N22°31’52.97"	E109°11’17.15"	74
*M*. *nana*	YNFM	Fuminxian, Yunnan, China	N25°18’21.31"	E102°41’24.88"	1958
	YNXW	Xuanweishi, Yunnan, China	N27°2’55.94"	E101°68’04.88"	2341
	YNWS	Wenshanxian, Yunnan, China	N23°19’27"	E103°13’34"	2583
	YNPL	Panlong, Kunming, Yunnan, China	N25°10’12.94"	E102°47’2.33"	2050
	YNHK	Haikouzhen, Kunming, Yunnan, China	N24°48’33.77"	E102°37’25.29"	1965
	YNZJ	Zhaojiaocun, Kunming, Yunnan, China	N25°05’13.64"	E102°34’10.37"	1888
*M*. *esculenta*	YNAL	Ailaoshan, Yunnan, China	N24°32’25"	E101°01’36"	2504
	YNWS	Wenshanxian, Yunnan, China	N23°19’27"	E103°13’34"	2583
	SCYB	Yanbianxian, Sichuan, China	N27°06’08.62"	E101°46’01.19"	2074
	SCMY	Miyixian, Sichuan, China	N27°02’55.94"	E101°58’04.88"	1952
*M*. *pensylvanica*	USBH	Harvard campus, Cambridge, MA, USA	N42°22’37.21"	W71°06’59.98"	8
*M*. *cerifera*	USR	NCSU campus, Raleigh, NC, USA	N35°47’04.79"	W78°40’55.54"	127
*Comptonia peregrina*	USR	NCSU campus, Raleigh NC, USA	N35°47’04.79"	W78°40’55.54"	127

### Acquisition and sequencing of the RAD libraries

Library preparation and sequencing of RAD markers from genomic DNAs were performed by Beijing Genomics Institute (Shenzhen, China) using the restriction enzyme *EcoR*I and sample-specific barcodes. The 18 individuals studied were first pooled and run in a single lane of an Illumina HiSeq 2000 with read length of 100bp, after which one individual of *C*. *peregrine* was sequenced for quality check, making 19 samples in total.

### De novo assembly

To process the raw RAD-seq data for phylogenetic analysis, we utilized the pyRAD software [15]. Given one or more Ilumina sequence files in FASTQ format, pyRAD can de-multiplex the data and create separate files for each sample according to their special barcode. We usually filtered sequences through the following steps: First, sequences containing sequencing errors in the cut site were discarded. Second, reads containing sequencing errors in the sample-specific barcode were removed. The restriction site and barcode were then trimmed from each sequence. Bases with a FASTQ quality score below a given value (here, 33) were replaced with N, sequences having more than a given percentage of Ns (here, 1%) were discarded. Paired-end reads of the same species were pooled together and de novo contigs were assembled using Trinity Release v2.0.6 [36], run with a kmer length of 25bp, set the minimum contig size as 150bp and with other parameters set to default.

### SNP discovery and phylogeny inference

To explore SNP markers for phylogenetic studies, we employed the pyRAD software, applied only to the single end of the paired-end sequences (R1). Because of the lack of a reference genome, sequence similarity is the simplest way to infer orthology. For each sample, sequences were clustered by similarity (here, 92%) using the uclust function in USEARCH [37] with heuristics turned off, yielding clusters representing putative loci. In order to ensure accurate base calls, clusters of fewer sequences than a set minimum depth of coverage (here, 6) were removed. The remaining clusters were then processed within pyRAD to generate consensus sequences. In pyRAD, the heterozygosity (H) and the error rate (E) are jointly estimated from the observed base counts across all sites in all clusters, by applying the maximum-likelihood equation of [38]. The mean E is then used to assign consensus diploid genotypes for each site in each cluster by calculating the binomial probability that the site is homozygous versus heterozygous given the relative frequencies of observed bases at the site and E [39]. If a base cannot be assigned with more than 95% probability, it is replaced by N in the consensus sequence. Heterozygotic variation is recorded using appropriate ambiguity codes.

Consensus sequences from all samples were clustered according to the sequence similarity using the same similarity threshold as in the previous step of within-sample clustering. For each cluster, sequences were aligned with Muscle v3.8.31 [40] with default parameters set. In order to detect potential paralogs, we set the maximum number (here, 3) of shared polymorphic sites in a locus, under the assumption that a shared heterozygous site across many samples likely represents clustering of paralogs with a fixed difference rather than a true heterozygous site [41]. The remaining clusters were treated as RAD loci and assembled into phylogenetic data matrices. For any given RAD locus, sequences of one or more samples may be missing if substitutions in the restriction site have disrupted recognition, or if the locus did not receive sufficient coverage for confident basecalling. To avoid the effect of missing data or insufficient information sites for phylogeny inference, the minimum taxa coverage (here, 12) of ingroup samples with data for a given locus to be retained in the final data set.

All phylogenetic analyses were conducted using the maximum-parsimony (MP), maximum-likelihood (ML) and Bayesian methods. Maximum-parsimony analyses were executed in PAUP* version 4.0b10 [42] with command files for the parsimony ratchet [43] generated using the program PRAP2 [44]. The following options were implemented: tree bisection-reconnection (TBR) branch swapping, characters treated as equally weighted and unordered, gaps treated as missing characters, and bootstrap analysis was performed with 10000 replicates. Maximum-likelihood method was implemented in RAxML-HPC v8.1.11 on the CIPRES cluster (http://www.phylo.org/) [45] using the general time-reversible (GTR) model of nucleotide substitution with gamma distributed rate heterogeneity. Bayesian inference (BI) implemented in Mrbayes v3.2.3 [46] using the best-fit model (GTR+G) according to the akaike information criterion (Posada & Buckley. 2004). Two independent parallel runs of four Metropolis-coupled Monte Carlo Markov Chains (MCMCs) were run with trees sampling every 1000 generations for five million total generations.

### Divergence time estimation

We first determined whether the aligned sequences were saturated for substitutions by performing the saturation test implemented in DAMBE [47]. The results of this test indicated no significant saturation signals. To calibrate our divergence date estimates of *M*. *rubra*, we set two normal priors. Firstly, the *Comptonia* node (node 1) was set to a minimum of 49 Ma (in Myr ago) based on *Comptonia columbiana*, which is a fossil species in the ‘Republic’ flora NE Washington and appears to be the oldest known Myricaceae fossil record [48]. Its leaves are easily recognized in the fossil record of Republic flora [49] that was dated back to 49 Ma using radiometric techniques [48]. Secondly, we estimated the split time between *M*. *esculenta* and *M*. *nana* and *M*. *rubra* based on data from the study of Myricaceae by Herbert [8]. The resulting time estimation (12.72±0.17 Ma) was set to the stem node of the *M*. *esculenta* (node 2). According to the two calibration points, divergence time of *M*. *rubra* was estimated under a Bayesian approach in BEAST v1.8.0 [50]. We implemented a Yule speciation tree prior and a GTR + G substitution model was selected as described above. MCMC runs were performed for 2 x 10^7^ generations, with sampling every 5000 generations, following a burn-in of the initial 10% cycles. Tracer v1.5 was used to examine the sampling adequacy and convergence of the chains to a stationary distribution. TreeAnnotator v2.0.2 was used to summarize the post burn-in trees and produce a maximum clade credibility chronogram showing mean divergence time estimates with 95% HPD intervals.

### Sequence annotation

In the following analysis, we removed three ingroup species (*M*. *cerifera*, *M*. *pensylvanica* and *M*. *adenophora*), of which only one sample was collected and thus generated insufficient read information. A BLASTX search was implemented against the NCBI non-redundant (Nr) protein database using BLAST, version 2.2.26 [51] with an E-value cut-off of 1e^-5^ for the contigs de novo assembled from each species. According to the results of the Nr protein database annotation, Blast2GO [52] was applied to obtain the functional classification of the contigs by following gene ontology (GO) terms (http://www.geneontology.org) [53], which maps contigs to function according to three principal GO categories: molecular function, cellular component and biological processes [54]. The results of the GO classification plot were obtained by WEGO (http://wego.genomics.org.cn/cgi-bin/wego/index.pl) [55]. The GO annotations of the contigs were mapped to the plant-specific GO slim ontology (http://www.geneontology.org/GO.slims.shtml) and the KEGG (http://www.genome.jp/kegg/) database.

## Results

### RAD tag generation and de novo assembly

After barcode trimming, cleaning and quality filtering, we obtained a total of 24.47 million paired-end reads (R1 = 78bp and R2 = 90bp). The sequencing quality was high; the Q33 of each sample was above 97%. The mean GC content of each sequence for the three species was c. 38.7% lower than the value of cDNA in the *M*. *rubra* cultivar ‘Biqi’ (49.65%) [22]. Detail information of RAD-tags sequencing is given in Table 2. De novo assembly was implemented in Trinity using R2 reads (without cut site), we obtained from 21902 to 29378 contigs with mean size of 229 to 234bp for *M*. *rubra*, *M*. *nana* and *M*. *esculenta*.

**Table 2:** Detail information of RAD-tag sequencing.

	*M*. *rubra*	*M*. *nana*	*M*. *esculenta*
Number of reads (million)	8.73	8.83	6.91
Total length of reads (million bp)	733	741	580
GC Rate %	38.29	39.08	38.72
Number of contigs	29378	21902	23552
Average contig length (bp)	229	234	234
N50	12705	9440	10150
Contig length range (bp)	150–472	150–414	150–442

### Phylogeny inference and divergence time estimation

**Fig 2:**
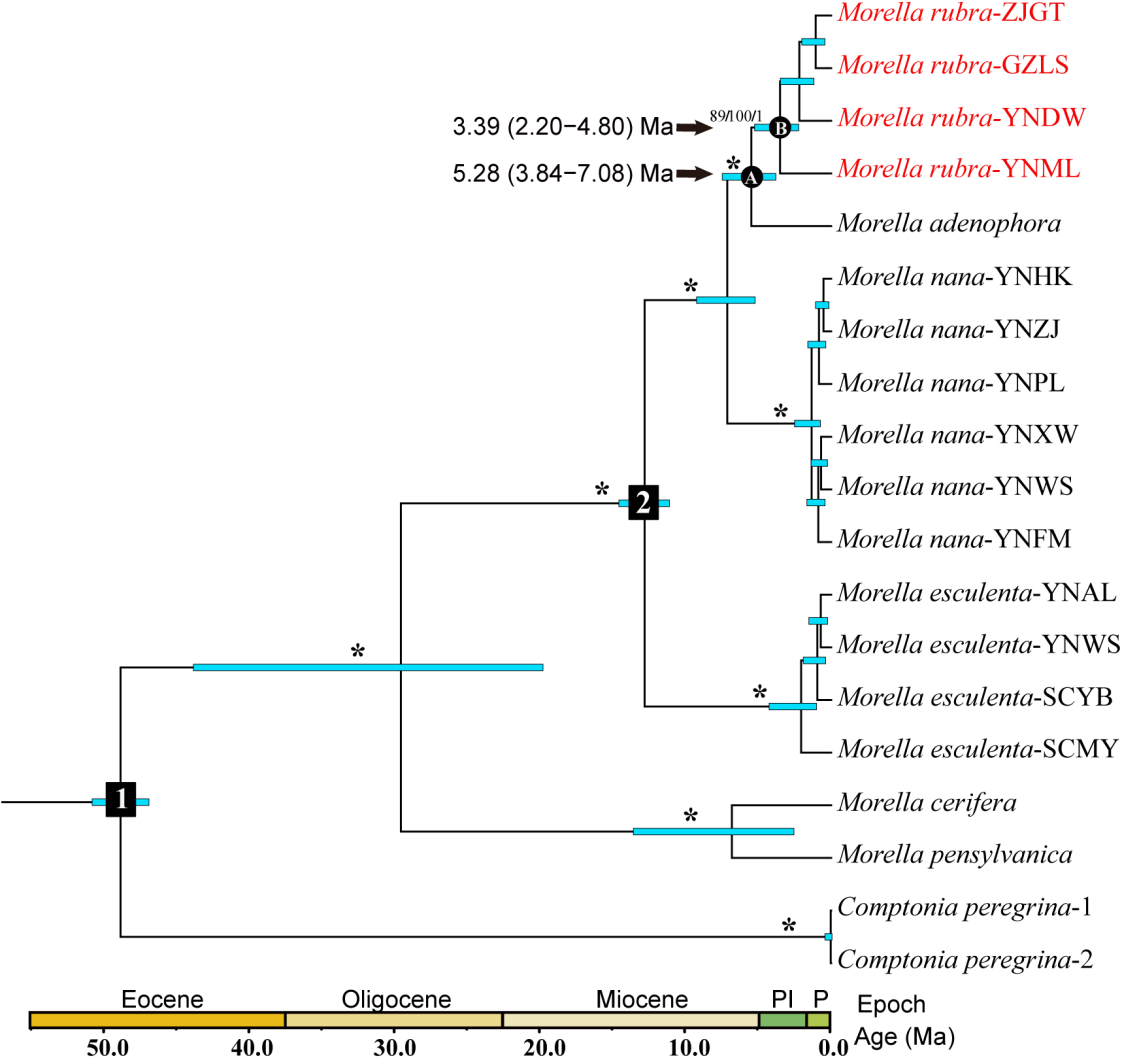
Bayesian phylogeny and divergence time estimation of *Morella*. Node1 and node2 represent two calibration points described in methods above. Blue bars indicate the 95% highest posterior density (HPD) credibility intervals for node ages (Ma). Asterisk indicates that maximum-parsimony bootstrap/maximum-likelihood bootstrap/Bayesian inference posterior probability equal to 100/100/1.

The single end of the paired end reads (R1) was applied for Phylogeny inference and divergence time estimation. We recovered c. 0.95×10^6^ reads from each sample of Illumina sequencing. After filtering and clustering at 92% similarity with coverage greater than 6, we obtained c. 64,129 clusters per sample with a mean depth of 10.44. Around 33631 consensus loci passed filtering for paralogs (Table 3). The sequencing error (E = 1.63×10^−3^) was lower than heterozygosity (H = 8.18×10^−3^) by ML estimation. After clustering of consensus sequences across all 19 samples under the minimum taxa data set, we recovered 4253 loci including 8360 parsimony informative variable sites with 3677 unlinked SNP sites, which were applied for phylogeny inference of four species of *Morella* in China. For the Bayesian analysis, phylogeny reconstruction using the minimum taxa data set revealed strong support for the four species as a clade (1.00 PP), as well as the monophyly of each species. The topology of (((*M*. *rubra*, *M*. *adenophora*), *M*. *nana*), *M*. *esculenta*) was significantly favored. Two species from North America (*M*. *cerifera* and *M*. *pensylvanica*) were sister to the four Chinese species. The tree topologies from the MP and ML analyses (S1 Fig) were consistent with the results of the Bayesian analysis (Fig 2).

**Table 3:** Results of filtering and clustering of one single end RAD sequences (R1) from 19 samples in this study.

Taxon	ID	RAD tags (×10^6^)	Clusters at 92%^1^	Mean depth	Consensus loci^2^	Number of loci in the minimum-taxa
*Morella rubra*	ZJGT	1.38	99748	11.19	67569	4100
	GZLS	1.11	91632	9.81	54655	3742
	YNDW	0.90	70869	9.63	33965	3197
	YNML	0.99	78684	9.87	43887	3621
*M*. *adenophora*	GXHX	0.71	41305	10.04	17345	2855
*M*. *nana*	YNFM	0.88	49315	10.65	22532	3255
	YNXW	1.09	66835	11.26	38145	3852
	YNWS	0.86	57805	9.67	26756	3375
	YNPL	0.85	52608	10.53	25070	3490
	YNHK	0.72	41091	10.86	16550	2781
	YNZJ	1.09	89046	9.12	54454	3831
*M*. *esculenta*	YNAL	0.77	52842	9.82	22825	2961
	YNWS	0.90	64529	10.24	32974	3504
	SCYB	0.88	62287	10.44	32872	3591
	SCMY	0.91	65235	10.19	35229	3675
*M*. *pensylvanica*	USBH	1.00	69555	10.82	38171	2722
*M*.*cerifera*	USR	1.01	60548	10.45	29835	2330
*Comptonia Peregrine*-1	USR1	0.96	43671	11.61	17602	1065
*Comptonia Peregrine*-2	USR2	1.11	60849	12.18	28561	1230

Note: ^1^Clusters with more than the minimum depth of six reads.

^2^Consensus loci which passed filtering for paralogs.

The BEAST-derived RAD-taq chronogram of *Morella* (Fig 2) recovered the four individuals of *M*. *rubra* as monophyly (posterior probability, PP = 1.00), with an estimated stem and crown age were c. 5.28 Ma (95% HPD: 3.84–7.08 Ma, Node A) and c. 3.39 Ma (95% HPD: 2.20–4.80 Ma, Node B) respectively. For this chronogram, BEAST provided an average substitution rate of 9.40×10^−9^ s/s/y, which is congruent with the mean values reported for plant nuclear DNAs (5.0–30.0×10^-9^s/s/y) [56]. Divergence time estimation indicated that the origin of *M*. *rubra* was near the Miocene-Pliocene boundary; intraspecific divergence in *M*. *rubra* occurred in the late Pliocene.

After clustering of the consensus loci for four individuals (GTS, GZLS, DWS and MLP), we identified 3808 unlinked SNP sites within the populations of *M*. *rubra*. These SNP markers may be applicable in future studies of the population genetics of *M*. *rubra*.

### Sequence annotation and GO enrichment analysis

**Fig 3:**
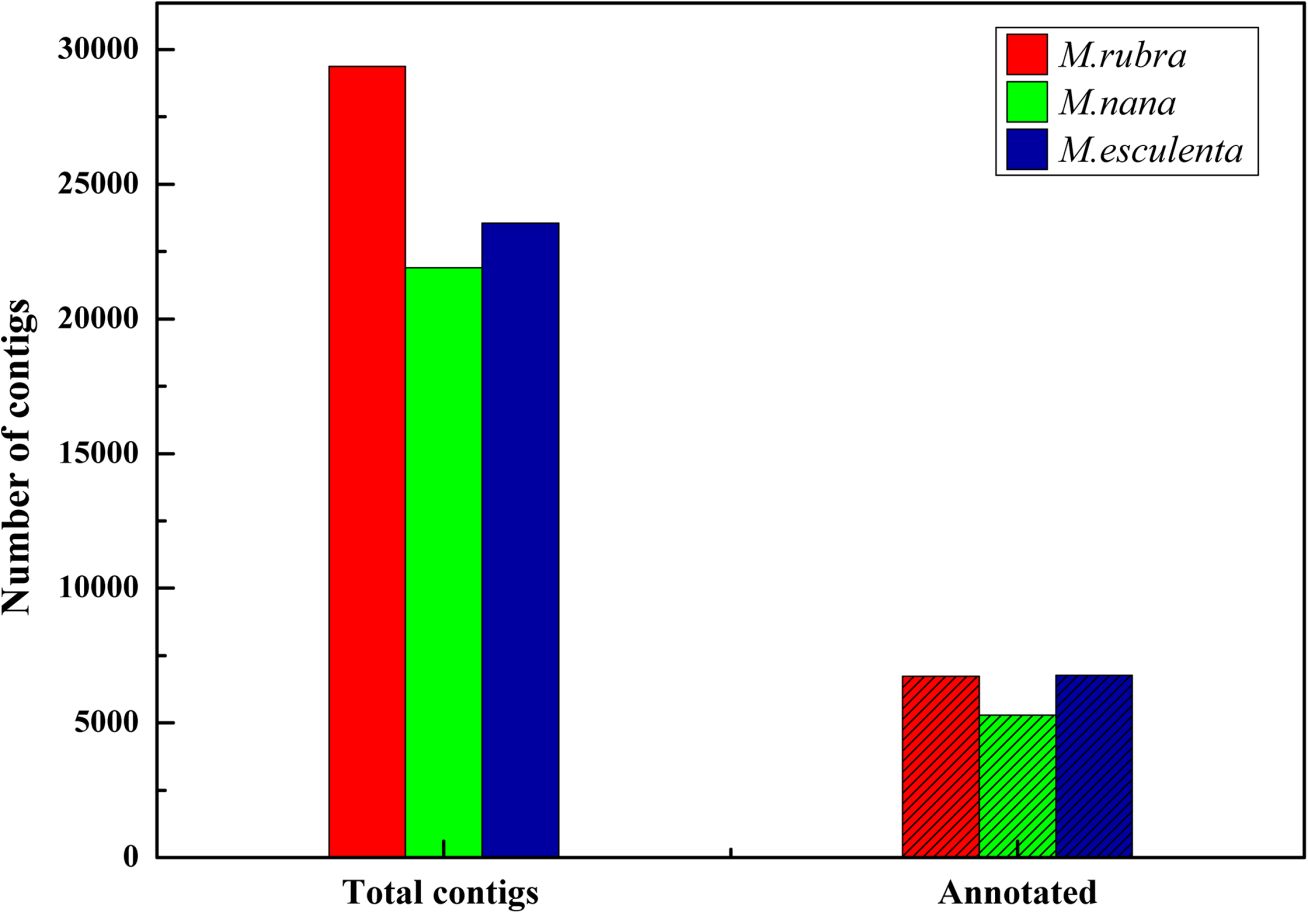
Number of the annotated contigs.

**Fig 4:**
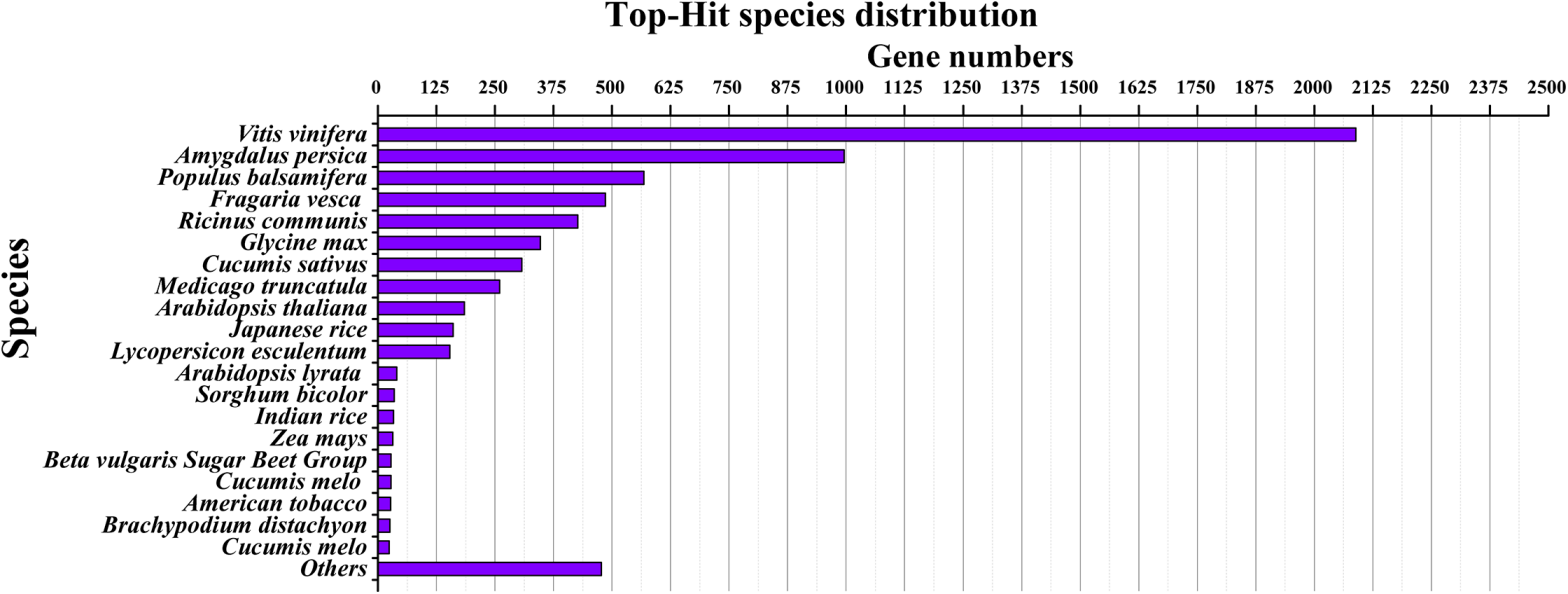
Top hit species distribution of *M. rubra* for BLAST result.

Based on the public Nr databases, 22.9% (6730) of assembled contigs in *M*. *rubra*, 24.1% (5287) in *M*. *nana* and 28.7% (6769) in *M*. *esculenta* were definitely mapped to known genes (Fig 3). The summary of the annotated contigs function is described in S1 Table. The top-hit species distribution of *M*. *rubra* for BLAST results were as follows: *Vitis vinifera*, *Amygdalus persica*, *Populus balsamifera*, *Fragaria vesca*, *Ricinus communis*, *Glycine max* and *Cucumis sativus* (Fig 4). *M*. *nana* and *M*. *esculenta* received nearly the same results (S2 Fig).

**Fig 5:**
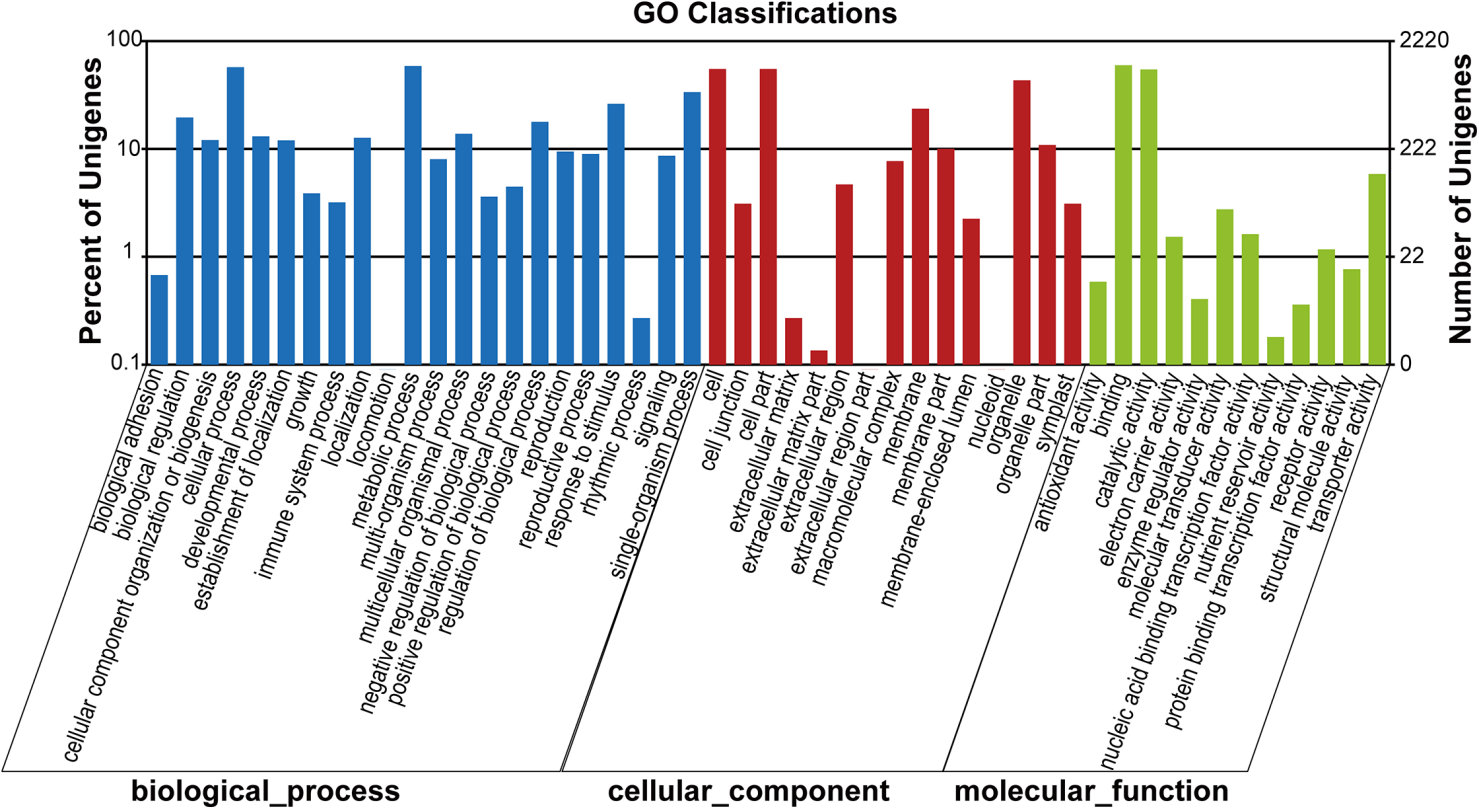
GO classifications of annotated contigs of *M. rubra*.

According to the results of Nr protein database annotation, 6730, 5287, and 6769 contigs for *M*. *rubra*, *M*. *nana* and *M*. *esculenta* respectively were implemented in Blast2GO for functional classification. For *M*. *rubra*, genes involved in cellular processes (GO: 0009987) and metabolic processes (GO: 0008152) are the top two most abundant subcategories in the biological process. Cell (GO: 0005623) and Cell part (GO: 0044464) are highly represented under the cellular component category. Binding (GO: 0005488) represents the major proportion of molecular function (Fig 5). *M*. *nana* and *M*. *esculenta* showed similar results (S3 Fig).

Chinese bayberry is generally thought to have been domesticated from the wild *M*. *rubra*. The lack of detailed knowledge of the genetics of wild populations is seriously hindering the industry’s ability to improve commercial stocks. To remedy this situation, we obtained annotated contigs of *M*. *rubra* to map to the KEGG database and identified those that may be correlated with fruit ripening, senescence, fruit quality and disease/defense (Table 4, S2 Table).

**Table 4:** Annotated contigs associated with fruit ripening and senescence, fruit quality formation and disease/defense metabolism in *Morella rubra*.

	Kegg ID	Deduced protein	Pathway involved
**Ripening and senescence**			
	K14516	ethylene-responsive transcription factor 1	Plant hormone signal transduction
	K14517	ethylene-responsive transcription factor 2	Plant hormone signal transduction
	K14514	ethylene-insensitive protein 3	Plant hormone signal transduction
	K05933	aminocyclopropanecarboxylate oxidase	Cysteine and methionine metabolism
	K00434	L-ascorbate peroxidase	Ascorbate and aldarate metabolism
	K01365	cathepsin L	Lysosome and Phagosome
**Quality formation**			
Color			
	K05280	flavonoid 3'-monooxygenase	Flavonoid biosynthesis
	K09422	myb proto-oncogene protein, plant	
	K12930	anthocyanidin 3-O-glucosyltransferase	Anthocyanin biosynthesis
	K12338	anthocyanidin 3-O-glucoside 5-O-glucosyltransferase	Anthocyanin biosynthesis
Texture			
	K01213	galacturan 1,4-alpha-galacturonidase	Pentose and glucuronate interconversions
	K01184	polygalacturonase	Pentose and glucuronate interconversions
	K00430	peroxidase	Phenylpropanoid biosynthesis
Aroma			
	K00001	alcohol dehydrogenase	Degradation of aromatic compounds
	K15086	(3S)-linalool synthase	Monoterpenoid biosynthesis
	K14174	beta-farnesene synthase	Sesquiterpenoid and triterpenoid biosynthesi
	K15797	(S)-beta-macrocarpene synthase	Sesquiterpenoid and triterpenoid biosynthesi
	K15799	(+)-alpha-barbatene/beta-chamigrene/thujopsene synthase	Sesquiterpenoid and triterpenoid biosynthesis
**Disease/defense**			
	K13456	RPM1-interacting protein 4	Plant-pathogen interaction
	K13457	disease resistance protein RPM1	Plant-pathogen interaction

## Discussion

### Phylogenetic relationships and taxonomy of *Morella rubra* and its close relatives

Previous phylogenetic studies have shown that *Myrica gale* and *M*. *hartwegii* are distinct from other species in the Linnaean genus *Myrica* [8, 57], therefore requiring recognition of two genera, *Myrica sensu stricto* (2 spp.) and *Morella* Lour.. Such a treatment is also supported by morphology [58] and cytology [59]. The four Chinese species were found to form a monophyletic clade in the *Morella*, but the phylogenetic relationships between them were not resolved [8], probably due to insufficient informative loci. In this study we used RAD-seq data to resolve the phylogenetic relationships of four species of *Morella* in China. In comparison to ITS and cpDNA markers, RAD-seq is excellent because more than 4253 loci including 8360 parsimony informative sites (the minimum-taxa data set) were generated. The phylogenetic relationships of the four Chinese species of *Morella* are well resolved with highest support. *M*. *esculenta* is basal in the Chinese *Morella* clade, which is congruent with the study by Hebert [8]. *M*. *nana* formed a strongly supported clade that is sister to the (*M*. *adenophora* + *M*. *rubra)* clade. Our result showed RAD-seq to be an effective approach for resolving phylogenetic relationships among closely related species.

In regards to the taxonomy, the four *Morella* species in China are easily distinguished with each other by the habit, indumentum, inflorescence type, fruit shape, flowering season, leaf, and flower morphology [9]. This agrees with our RAD-seq based phylogeny. So far, there are still many remaining controversies in *Myrica* species in the Indo-China region. In addition, many of these species are actually *Morella*, but most of the names have not been transferred to the right genus yet. For example, one (*Myrica esculenta* Buch.-Ham. ex D. Don) to five species (*M*. *esculenta*, *M*. *farquhariana* Wall., *M*. *sapida* Wall., *M*. *nagi* Thunb., *M*. *integrifolia* Roxb.) are recognized in India by different authors. Yanthan et al. [60] tried to resolve this dispute using the 18S-26S rDNA ITS sequences and proposed that *M*. *nagi* and *M*. *esculenta* should be treated as two separate species. This effort helps us to understand the complexity of *Myrica* species in Indo-China region. However, we believe that a phylogenetic study based on next-generation sequencing (such as RAD-seq in this study) and more comprehensive sampling will easily solve the mystery that can finally provide us a natural classification system for Myricaceae.

### Intraspecific divergence in *M. rubra* driven by the third uplift of the QTP

The origin and evolution of biodiversity is always linked with a range of geological or climatic processes, such as continental drift, the uplift of mountain chains and climatic fluctuation associated with ice ages. These processes can create new habitats and provide opportunities for speciation by interacting with each other [61]. Historical orogenesis and climatic oscillations can also cause fragmentation of species distributions and isolation of populations, leading to reduced gene flow and allopatric divergence [62]. To date, a series of studies have shown that ecological factors, such as temperature and precipitation, can drive speciation or infraspecific divergence [63–65]. Based on RAD-seq data, we determined that speciation of *M*. *rubra* was at c. 5.28 Ma (95% HPD: 3.84–7.08 Ma), and infraspecific divergence in *M*. *rubra* occurred in the late Pliocene (3.39Ma, 95% HPD: 2.20–4.80 Ma). The latter timescale is consistent with the third intense uplift of the Qinghai-Tibet Plateau (QTP) and the formation of the Hengduan Mountains (c. 3.6 Ma) [66–67]. Our ongoing study on the domestication of *M*. *rubra* also reveals that wild populations from Yunnan are the basal ones, indicating that it might have originated in the Hengduan Mountains area (unpublished data). Therefore, it is likely that intraspecific divergence in *M*. *rubra* occurred in the late Pliocene, and was driven by the uplift of the QTP and the formation of the Hengduan Mountains.

### Valuable genetic resources of Chinese bayberry based on RAD-seq

Chinese bayberry is a specialty fruit of China, and grown commercially in eastern and western China. It is one of the most popular fruit crops because of its food, medicinal and landscape value and has become an important export product in China [68]. However, it is highly perishable and susceptible to mechanical injury, physiological deterioration and fungal decay, resulting in a postharvest life of only 1 to 2 days under ambient temperature [31].

Feng et al. [22] analyzed the RNA-seq of Chinese bayberry to determine the molecular mechanisms for change in fruit color and taste during ripening. Zhu et al. [26] analyzed 2000 EST sequences from the cDNA libraries of Chinese bayberry cultivar ‘Biqi’, and identified several genes associated with disease/defense and anthocyanin accumulation, gene encoding elements correlated with ethylene biosynthesis and signal transductions, and proteins linked to senescence regulation and quality during fruit ripening. In this study, based on de novo assembly using R2 reads, we identified annotated contigs which are thought to be correlated with fruit ripening and senescence, fruit quality, disease/defense metabolism, and other important pathways.

Chinese bayberry has been cultivated for more than 2000 years, but detailed studies of its biology started only three decades ago [25]. There are approximately 305 recorded accessions, of which 268 are named cultivars [69]. Zhang et al. [25] used amplified fragment length polymorphism (AFLP) to reveal genetic diversity of 100 accessions of Chinese bayberry, and showed that the subgroups were somewhat related to the region of origin of the accessions, but accessions from the same region did not necessarily belong to the same group or subgroup due to extensive gene flow among different regions. Jiao et al. [24] developed simple sequence repeat (SSR) markers for Chinese bayberry and came to a similar conclusion. However, none of the previous studies was able to reveal the relationships among cultivars with high resolution, probably because of an insufficient number of informative sites. Besides, studies on genetic diversity and population structure of wild *M*. *rubra* populations are limited. The origin of domesticated Chinese bayberry has never been resolved. In our study, however, 3808 SNPs were identified within wild *M*. *rubra* populations, which will be a valuable resource for subsequent studies on the population genetics of *M*. *rubra* and the domestication of Chinese bayberry.

## Supporting Information

S1 Fig Phylogeny inference using Maximum parsimony (MP) method and Maximum likelihood (ML) methods. (TIFF)

S2 Fig The top hit species distribution of *M. nana* and *M. esculenta* for BLAST result. (TIFF)

S3 Fig GO classifications of annotated contigs of *M. nana* and *M. esculenta*. (TIFF)

S1 Table Top BLAST hits from public databases. Lists of the top results from BLASTING *M. rubra*, *M. nana* and *M. esculenta* contigs against public databases (E-value cut-off of 10^−3^).
(XLSX)

S2 Table The result of the annotated contigs against KEGG database for *M. rubra*. (XLS)
