# Supplementary figures and images for: Phylogeny of Morella rubra and Its Relatives (Myricaceae) and Genetic Resources of Chinese Bayberry Using RAD Sequencing
Source: PLoS One. 2015 Oct 2;10(10):e0139840. doi: 10.1371/journal.pone.0139840 (PMC4591994; doi:10.1371/journal.pone.0139840)

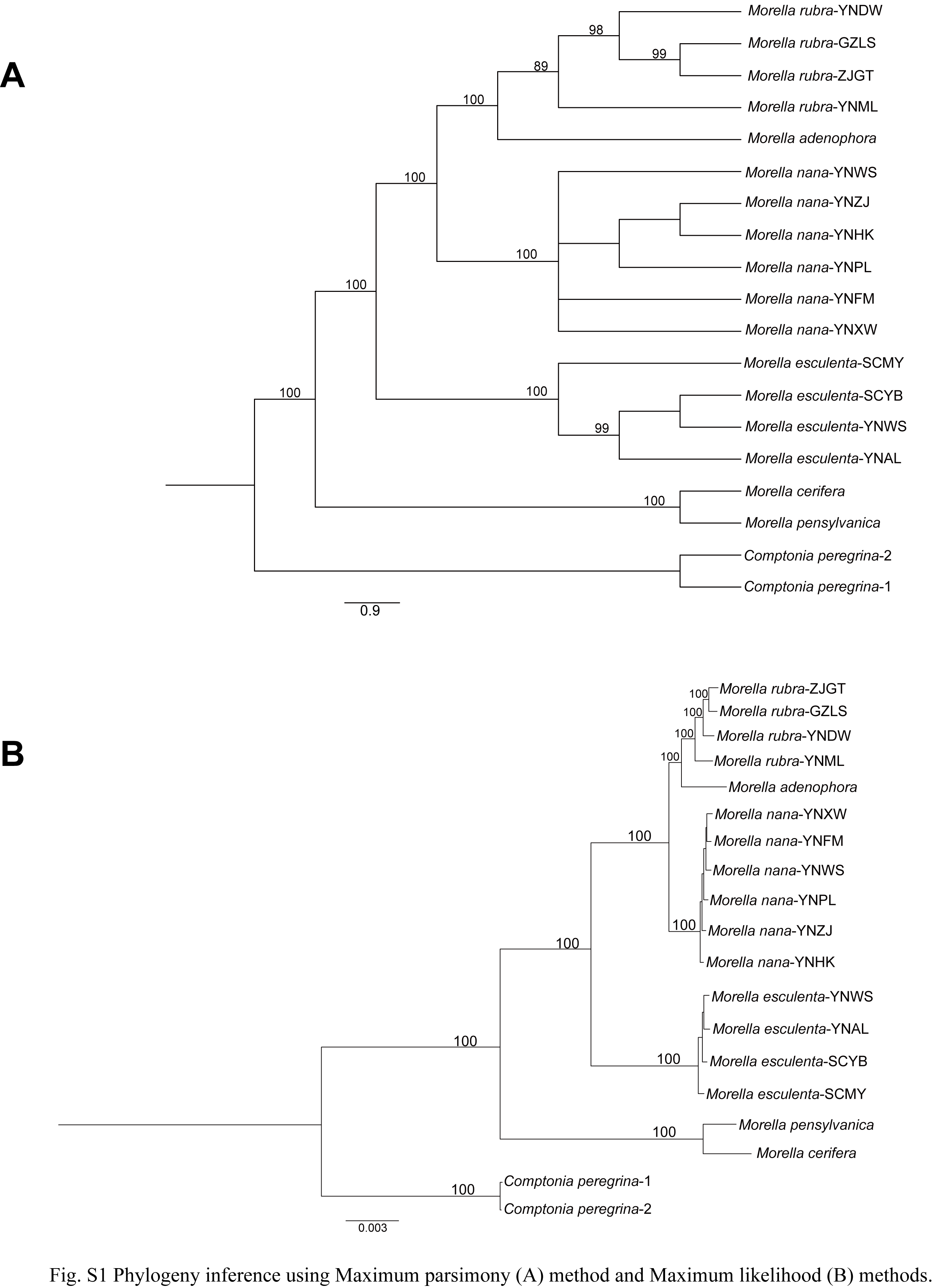

Supplement: S1 Fig — (TIFF) [file pone.0139840.s001.tiff]

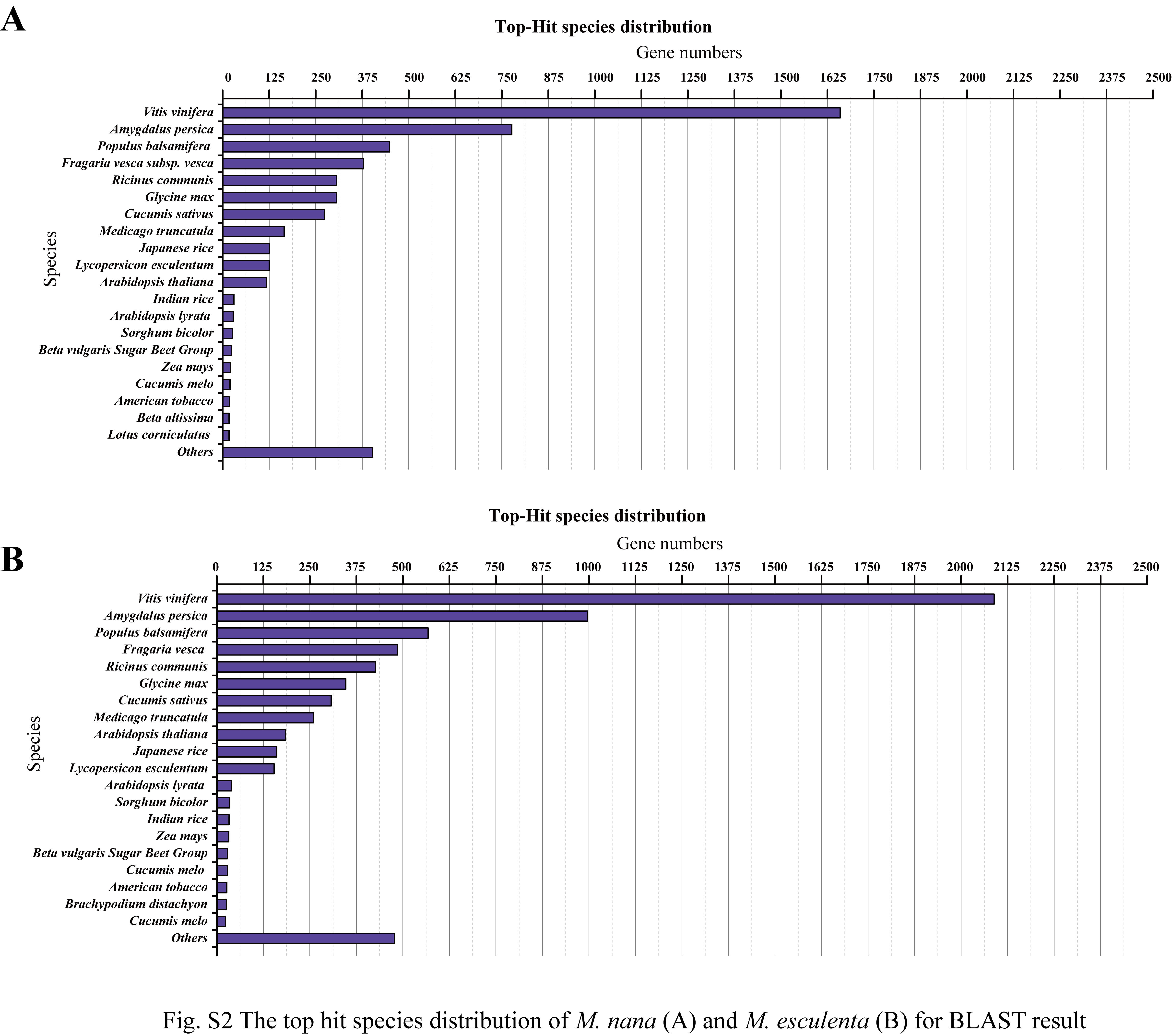

Supplement: S2 Fig — (TIFF) [file pone.0139840.s002.tiff]

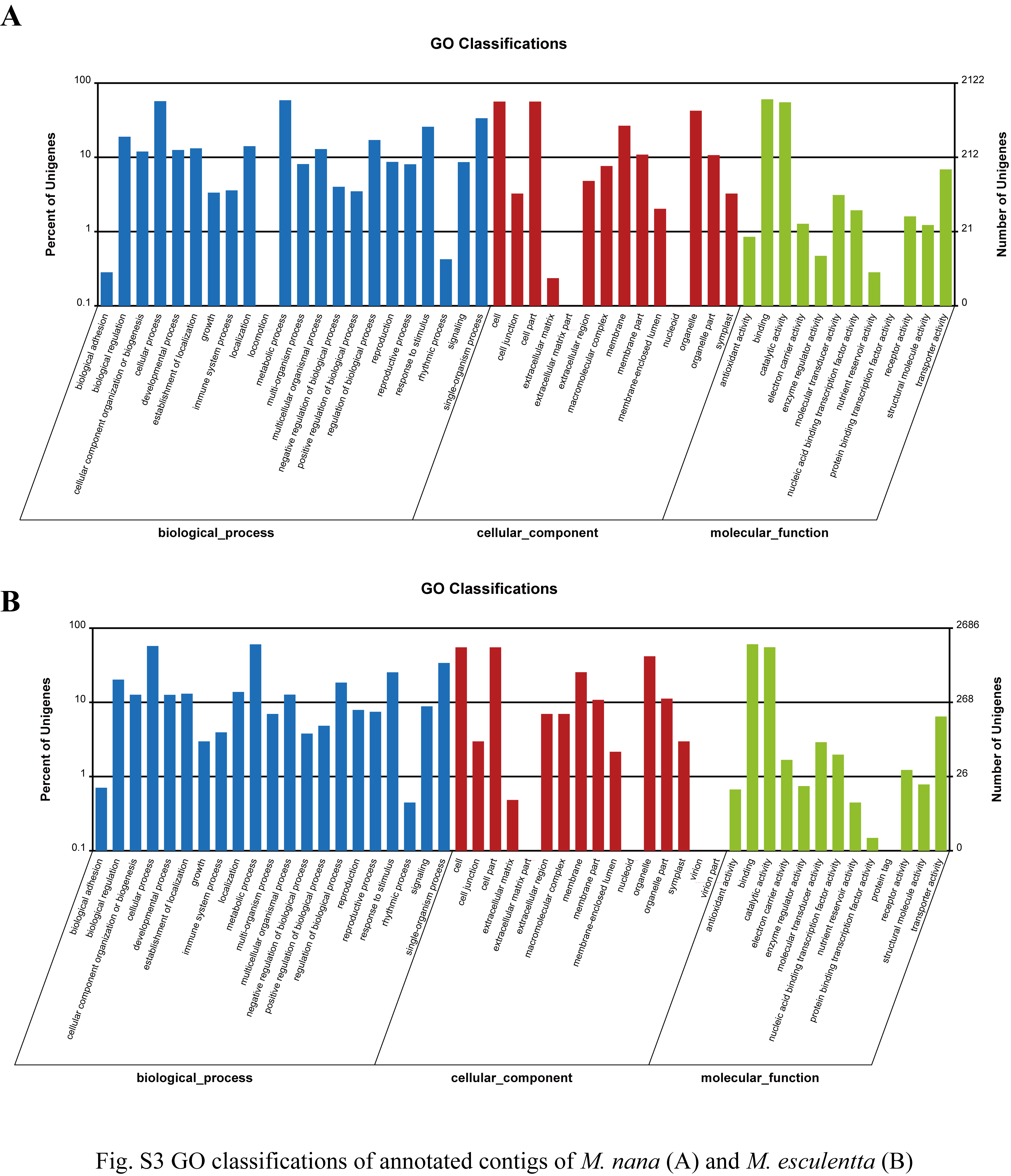

Supplement: S3 Fig — (TIFF) [file pone.0139840.s003.tiff]
